# Microfluidic-Based Novel Optical Quantification of Red Blood Cell Concentration in Blood Flow

**DOI:** 10.3390/bioengineering9060247

**Published:** 2022-06-08

**Authors:** Yudong Wang, Bharath Babu Nunna, Niladri Talukder, Eon Soo Lee

**Affiliations:** 1 Advanced Energy Systems and Microdevices Laboratory, Department of Mechanical and Industrial Engineering, New Jersey Institute of Technology, Newark, NJ 07102, USA; yw35@njit.edu (Y.W.); nt22@njit.edu (N.T.); 2 Department of Mechanical Engineering, Weber State University, Ogden, UT 84408, USA; bnunna@weber.edu

**Keywords:** microfluidics, biosensing platform, hematocrit quantification, optical measurement, grayscale

## Abstract

The optical quantification of hematocrit (volumetric percentage of red blood cells) in blood flow in microfluidic systems provides enormous help in designing microfluidic biosensing platforms with enhanced sensitivity. Although several existing methods, such as centrifugation, complete blood cell count, etc., have been developed to measure the hematocrit of the blood at the sample preparation stage, these methods are impractical to measure the hematocrit in dynamic microfluidic blood flow cases. An easy-to-access optical method has emerged as a hematocrit quantification technique to address this limitation, especially for the microfluidic-based biosensing platform. A novel optical quantification method is demonstrated in this study, which can measure the hematocrit of the blood flow at a targeted location in a microchannel at any given instant. The images of the blood flow were shot using a high-speed camera through an inverted transmission microscope at various light source intensities, and the grayscale of the images was measured using an image processing code. By measuring the average grayscale of the images of blood flow at different luminous exposures, a relationship between hematocrit and grayscale has been developed. The quantification of the hematocrit in the microfluidic system can be instant and easy with this method. The innovative proposed technique has been evaluated with porcine blood samples with hematocrit ranging from 5% to 70%, flowing through 1000 µm wide and 100 µm deep microchannels. The experimental results obtained strongly supported the proposed optical technique of hematocrit measurement in microfluidic systems.

## 1. Introduction

Microfluidic biosensor platforms can be used to diagnose diseases with high accuracy, however, the sensing mechanisms are critically impacted by hematocrit (Hematocrit), the volumetric percentage of red blood cells (RBCs) [1]. It is a critical hematological index for the diagnosis of many diseases, for example a lower hematocrit suggests anemia and a higher hematocrit indicates dehydration [2,3,4]. The hematocrit is usually measured using the centrifugation or complete blood cell count methods (CBC) [5]. The blood sample is drawn into a capillary tube and centrifuged in the centrifugation method. After centrifugation, the packed red blood cells sediment to the bottom, and a ratio of RBCs and the whole blood can be measured from the centrifuge tubes [6,7,8,9]. In the CBC measurement process, the whole blood sample is diluted with an isotonic solution before the analysis. The blood sample is forced to flow between two electrodes. A constant current is applied to the electrodes to generate a consistent impedance, until the blood cells have passed between the electrodes. The RBCs are counted by the change in the electrical resistance in the circuit, as the RBC membrane is non-conductive. The hematocrit can be calculated from the RBC count, the mean blood cell volume, and the dilution factor [10,11]. Some other quantification methods developed for point-of-care testing (POCT) environments also have shown promising results, such as paper-based microfluidic devices, hemoglobin measurement, and so on [12,13,14,15,16]. The centrifugation, CBC, and many POCT hematocrit quantification methods are well-suited to measure the blood samples. However, these methods cannot measure the hematocrit in the blood flow in microfluidic systems. The hematocrit of the blood flow varies in the microchannel due to the hydrodynamic forces applied to the blood cells, especially in curved channels, where the blood cells can migrate significantly along the channel width [17,18,19]. The hematocrit at the different locations of the microchannel deviates from the blood sample’s hematocrit at the inlet and thus affects the sensor measurement accuracy, especially for the sensors embedded in the microchannel [20,21,22,23,24,25,26,27,28,29,30,31,32].

Besides measuring the hematocrit in a tube, it has been of huge interest to the POCT field to know the hematocrit change in microfluidic blood flow. Hematocrit plays a critical role in blood flow. It can significantly affect the viscosity of the blood [32,33]. In low-shear-rate blood flow, the hematocrit also influences the non-Newtonian property of the blood flow [34,35]. Therefore, it is essential to know how the hematocrit changes in microfluidic blood flow to better understand the fluid dynamics in microfluidic systems and enhance the performance of the biosensors [36]. However, the quantification methods in the blood sample preparation stage cannot account for the hematocrit changes in the flow motion. Recently, researchers developed a flow cytometry-based method to measure the hematocrit in microfluidic blood flow. However, the flow cytometry-based method is expensive, and the flow image can be blurry at a high flow rate without a high-speed camera [37]. Herein, a practical optical quantification method of the hematocrit in the microfluidic blood flow has been proposed.

As blood is a translucent fluid, it can be observed in the microchannel using an inverted transmission microscope, which is standard equipment in most laboratories. It consists of 55% plasma, 44% RBCs, 1% white blood cells (WBCs) and platelets [38]. The plasma is a pale yellow liquid in which the blood cells are suspended. RBCs mainly consist of two components: cell membrane and hemoglobin [39,40]. The heme in the hemoglobin has ferrous ions, which can combine with oxygen and appear in red [41]. When the blood supplies the oxygen to tissues, the heme is deoxygenated, and the hemoglobin turns darker [42,43,44]. WBCs constitute roughly 0.7% of the blood’s volume, and platelets are also less than 1%, so the contribution of WBCs and platelets to the overall color of blood is negligible [45]. Venous blood is the commonly used sample in laboratories, and it is deoxygenated blood. Deoxygenated blood (oxygen saturation 70% to 80%) absorbs more red light than oxygenated blood (oxygen saturation higher than 94%) and appears dark red [46,47]. 

For this experiment, deoxygenated blood samples from two donors were diluted/concentrated to various hematocrits ranging from 5% to 70% by adding/removing plasma. As the transparency of plasma and RBCs is different, the higher hematocrit blood has a higher volumetric concentration of RBCs and blocks more light transmission, reducing the light intensity received by the high-speed camera of the microscopy system. Because of this optical property, it is possible to measure the hematocrit of the blood from the light intensity detected by the camera sensor, quantified by the grayscale (a range of gray shades from black to white and values from 0 to 255, respectively) of the image taken by the camera [48]. A higher amount of light would be transmitted through the blood samples with lower hematocrit throughout the blood flow in the microchannel and shows a higher value in the grayscale of the images, and vice versa [49].

Though some research groups have investigated the quantification techniques of the hematocrit using grayscale image methodology, the lack of good reproducibility and complexity in implementing those methods hinders their techniques in practical applications. Gester et al. presented an investigation of the RBCs distribution of various hematocrit blood flows in microchannels with different diameters and flow rates by using normalized grayscale of the flow images. However, the light settings and the relationship between image grayscale and hematocrit were not detailed in this study [50]. Jalal et al. developed a smartphone-based hematocrit determination application using the grayscale with a maximum number of pixels of the blood image in similar optical conditions. However, the phone camera settings were not mentioned [51]. Browne et al. used grayscale analysis to evaluate their rapid blood separation technique, but this method cannot be widely applied in other microfluidic systems [52]. The proposed technical approach in this research work to quantify the hematocrit in the microfluidic blood flow has significant advantages compared to the conventional approaches in terms of the duration of the diagnosis, it minimizes the potential for blood contamination, and it can be applied to a wide domain of applications, including the analysis of the cellular dynamics during the blood flow.

In this study, light source intensity, color temperature of the light source, high-speed camera exposure time, and polydimethylsiloxane (PDMS) substrate thickness affect the grayscale of the blood flow images, and all of these variables were carefully considered and strictly controlled during the experiments. The light source intensity directly affects the illuminance on the PDMS microchannel surface. The light spectrum is varied with the color temperatures of different light sources. The exposure time of the high-speed camera proportionally affects the total amount of luminous flux that reaches the camera sensor. The PDMS substrate thickness has a minor effect on the blood flow image, as the PDMS is optically transparent from the near UV (Ultraviolet) up to NIR (Near Infrared) region of the spectra, yet it was still controlled as 2-mm thick in this study. A relationship between grayscale and hematocrit of the blood at different optical conditions is detailed very clearly in this paper, and the effects of the light source condition and the camera settings are discussed to provide enhanced reproducibility.

## 2. Materials and Methods

### 2.1. Optical Settings

**Figure 1:**
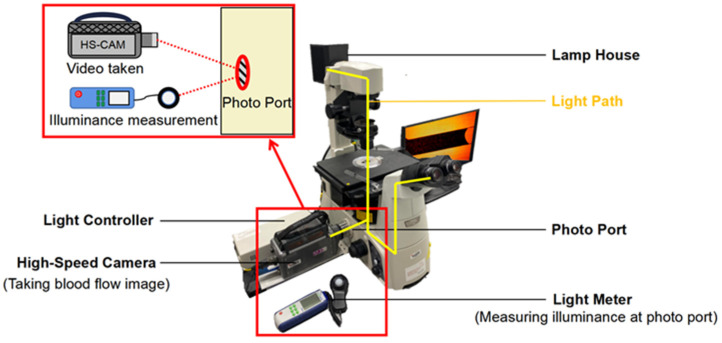
The schematic representation of the experimental setup was used to demonstrate the optical technique for the quantification of hematocrit.

Detailing the optical conditions while capturing the images of the blood flow plays a vital role in enhancing reproducibility. In the inverted transmission microscopy system, the light generated by the lamp house (100 V 12 W) must pass through the observing object (PDMS channel on a glass slide), objective lens, and reflective mirrors in this sequence and finally reach the camera sensor at the photo port. The PDMS was strictly controlled at 2 mm (±0.01 mm) thick in experiments to avoid any errors caused by different light transmissions resulting from various PDMS thicknesses. The luminous exposure is the total amount of luminous flux per unit area (lux) for a given time (µs) measured in lux·µs. It is vital that every image is taken in the same optical condition, as this linearly affects the average grayscale of the images of the blood samples with the same hematocrit. In this experimental setting, the illuminance was measured at the photo port of the microscope by a light meter, and the exposure time was set on the high-speed camera control software. Before measuring the illuminance, a glass slide with a clean 2 mm-thick PDMS piece was placed on the objective table and worked as a calibrator when measuring the illuminance. The illuminance at the photo port was adjusted by toggling the lamp house’s light intensity controller knob. In this way, the luminous exposure at the camera sensor can be known and fixed when taking the blood flow images. The microscopy system setup is shown in Figure 1.

### 2.2. Camera Settings

The following key camera settings were considered for capturing the images in this proposed novel optical technique: (1) the exposure time is the duration of time that the camera sensor is exposed to light for each frame in the video. It can linearly affect the luminous exposure at the camera sensor; (2) the white balance is a method to adjust the color filters of the digital camera to fit the actual color temperature of the light source. It needs to be adjusted with the color temperature of the halogen lamp house to have a more accurate color of the object. (3) Frame rate describes how many frames the camera can take in each second. It limits the exposure time of each frame but will not change the total light amount detected by the camera sensor.

### 2.3. Blood Samples Preparation

The experiments were conducted with porcine venous blood from two donors (procured from Lampire Biological Laboratories (Pipersville, PA, USA)), and on the same day that the blood was drawn to avoid red blood cell lysis and to ensure the accuracy of the data. The blood from each donor was centrifuged to separate the plasma and blood cells, and the hematocrit of each blood sample was measured using this technique. This process was repeated three times to calculate the average hematocrit percentage. Blood samples with various hematocrit percentages from 5% to 70% were prepared by adding or removing plasma to/from the blood. The sample preparation error was controlled by a micro-liter pipette and evaluated by the 0.1-milligram weight scale.

### 2.4. PDMS Microchannel Fabrication

**Figure 2:**
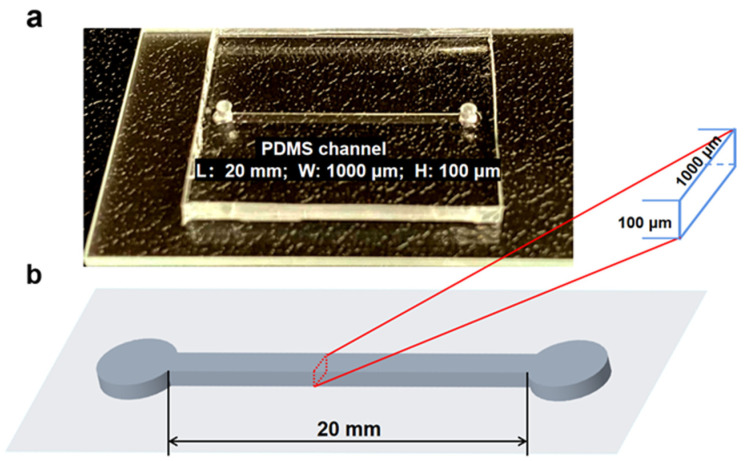
(**a**) PDMS microchannel 100 μm high and 1000 μm wide, with inlet and outlet ports. (**b**) Schematic drawing of the microchannel used in the experiments detailing the dimensions.

PDMS microchannels were fabricated by the soft lithography technique using a Si mold with microchannel structures. As a capillary flow-driven microfluidic channel, no external devices are needed to drive the blood flow in the microchannel [53,54]. PDMS is naturally hydrophobic. Thus, an oxygen plasma surface treatment is required to turn the PDMS surface hydrophilic before the introduction of the micro-liter blood droplet into the channel inlet. A 1000 µm wide and 100 µm deep microchannel was chosen for the experiments and is shown in Figure 2.

## 3. Experiment

### 3.1. Luminous Exposure Control

Before recording blood flow videos, a consistent optical condition needs to be well controlled. A higher or lower luminous exposure increases or decreases the average grayscale of the images with identical hematocrit blood, respectively. The luminous exposure values were controlled by changing the camera’s exposure time on the camera control software, with specific illuminances being measured by the light meter at the photo port of the microscope to ensure accurate measurement for each recording. The light meter (Digi-Sense WD-20250-00) with ±3% accuracy was purchased from Cole-Parmer. The luminous exposures from 2000 lux·µs to 21,000 lux·µs were well controlled in the experiments.

### 3.2. Capturing Blood Flow Images in the Microchannel

**Figure 3:**
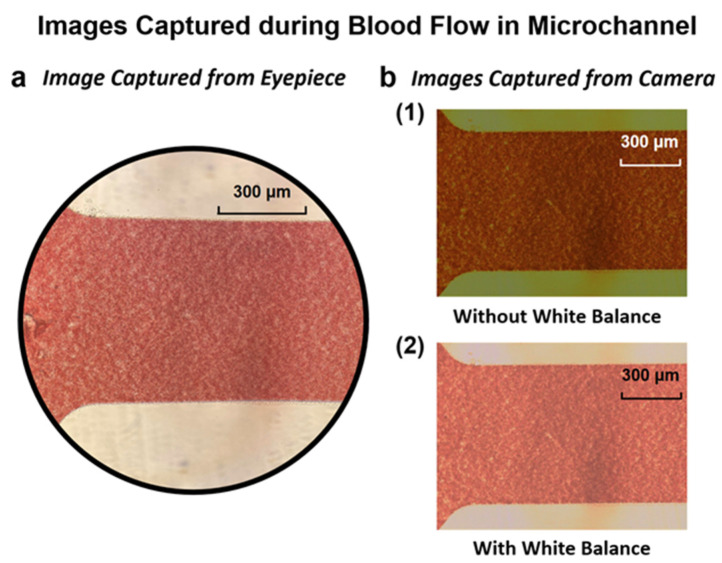
Comparison of images captured during the flow of blood with 15% hematocrit in the microchannel (**a**) from the eyepiece of the microscope and (**b**) from the camera (1) before white balance and (2) after white balance.

After setting the optical condition, a surface-treated 2 mm thick PDMS microchannel was placed on the objective table. Next, 5 µL of blood droplets with a particular hematocrit were introduced into the microchannel inlet using a micro-scale pipette. The 15% hematocrit blood flow was recorded using the appropriately white-balanced high-speed camera with a 500 fps frame rate. Figure 3 shows the white balance effect on the image quality, and the image of blood flow in the microchannel recorded by the camera. The above-mentioned procedures were repeated for each blood sample at each hematocrit and at every luminous exposure value to collect the full set of results.

### 3.3. Conversion of RGB Image to Grayscale Image

**Figure 4:**
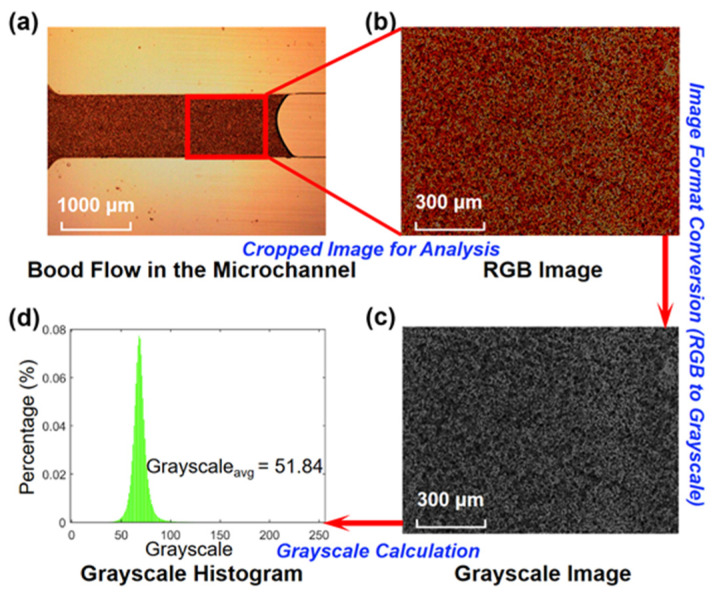
Schematic representation of the grayscale measurement to quantify the hematocrit during blood flow in the microchannel: (**a**) the frame captured from the video of the blood flow in the microchannel (with 1000 µm width and 100 µm depth); (**b**) cropped image from Figure 4a in RGB format; (**c**) converted grayscale image from Figure 4b; (**d**) the plot of the grayscale histogram with average grayscale of 51.84.

With constant illuminance at the microscope photo port and identical camera settings, the images of blood flow were shot and saved in 24-bit red, green, and blue (RGB) format. An RGB image is an additive color image mixed with red, green, and blue primary colors, and it contains both color and light intensity information. The RGB images were then converted to 8-bit grayscale images, and the color information was removed. Each pixel in grayscale images represents light intensity, and the value is scaled from 0 to 255 representing black (darkness) and white (brightness) shades, respectively. Figure 4 shows the average grayscale calculation process using the Matlab program. Using the concept of the average grayscale level of the image, each hematocrit blood sample image can be specified by the average grayscale at specific luminous exposures. Thus, the relationship between the hematocrit and average grayscale can be determined.

## 4. Results and Discussions

### 4.1. Luminous Exposure Effect on the Grayscale for Different Hematocrit Values

**Figure 5:**
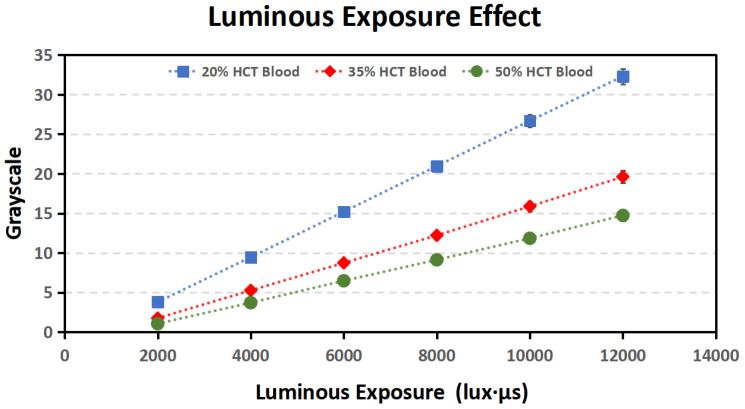
Plot of the luminous exposure vs. grayscale for blood samples with varied hematocrit of 20%, 35%, and 50%.

The luminous exposure linearly affects the average grayscale of the images taken by the camera. Figure 5 shows the linear relationships between luminous exposures and the grayscale of particular hematocrit values by measuring the grayscale of the images of blood flow with specific hematocrit values in the microchannel at different luminous exposure. The average grayscale levels of blood samples with 20%, 35%, and 50% hematocrit were measured from 2000 lux·µs to 12,000 lux·µs and showed a perfectly linear plot of the relationship between grayscale and luminous exposure.

### 4.2. Comparison of the Grayscale from the Samples of Different Donors

**Figure 6:**
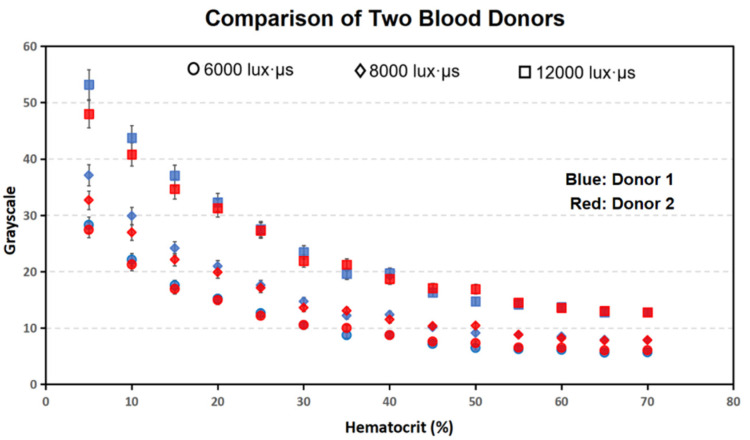
Comparison of the average grayscale of different hematocrit blood images from donor 1 (blue) and donor 2 (red) at 6000, 8000, and 12,000 lux·µs. Images of the blood from donor 1 and donor 2 were taken with 20 lux and 40 lux illuminance, respectively. Each data point takes the average of three technical replicates, and the error bar shows the standard deviation.

Figure 6 compares the average grayscale values for all of the images, at each of the different luminous exposures and hematocrit values, for both donor 1 and donor 2.

The plotted data shows a strong correlation at all luminous exposures. Moreover, the illuminance condition for donor 1’s images was 20 lux, and for donor 2’s images was 40 lux. The result confirms that (1) the relationship between the average grayscale and hematocrit of different porcine blood samples shows a correlates consistently under the same optical condition, which means the effects of the metabolic status of different donors can be negligible; and (2) controlling the luminous exposure allows for the collection of valid data in experiments, even with combinations of different illuminance and exposure times.

### 4.3. Results of Hematocrit and Grayscale at Different Ranges of Luminous Exposure

**Figure 7:**
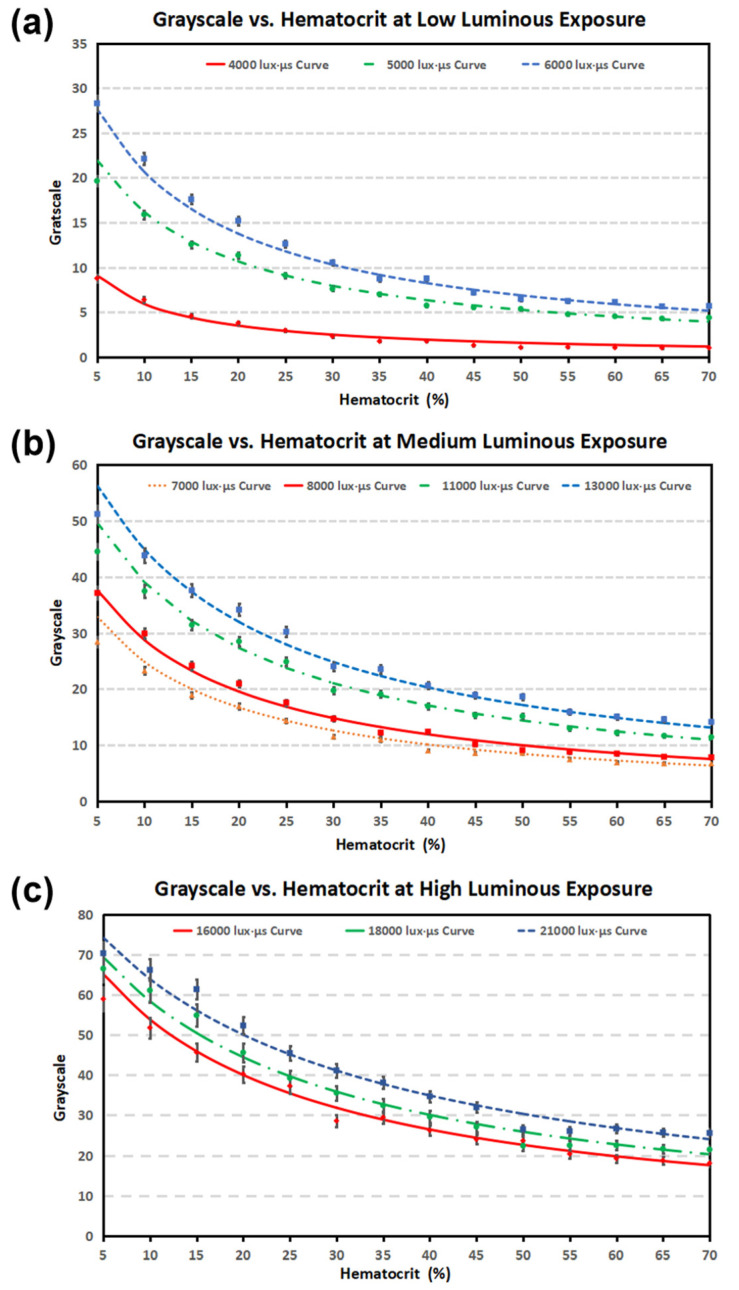
Plots of experimental results of grayscale vs. hematocrit of the blood at different luminous exposures (Hv) within (**a**) the low luminous exposure range (<6000 lux·µs), with an average R^2^ = 0.992 (the square of the correlation coefficient indicates that 99.2% of the variation in the grayscale can be explained by hematocrit), (**b**) the medium luminous exposure range (6000 lux·µs~15,000 lux·µs), with an average R^2^ = 0.990, and (**c**) the high luminous exposure range (>15,000 lux·µs), with an average R^2^ = 0.983. Each data point takes the average of three technical replicates.

Multiple light conditions were considered in this study, due to the different observational and video recording purposes. The luminous exposure range was reasonably selected to ensure the blood flow was visible in the microscope and recorded video. Figure 7 shows the relationship between the average grayscale and hematocrit of blood samples at different luminous exposures. When the optical condition is too dim or too bright, the color temperature will change and give blood images an inaccurate color appearance. Therefore, the luminous exposures were divided into low, medium, and high ranges.

The plotted curves are all rational, rather than linear. The color of the blood is always reddish, more concentrated blood appears darker, and more diluted blood is brighter. The average grayscale of the most concentrated blood (70% hematocrit in the experiment) cannot reach 0 (grayscale = 0 means pure black). A relation of RGB to grayscale conversion that is proposed in [55] is used in this Matlab computation as follows:

(1) Grayscale=0.299·R+0.587·G+0.114·B

where R, G, and B are the red, green, and blue color intensity values ranging from 0 to 255. The most diluted blood (5% hematocrit in the experiment) still appears lightly reddish, and can only reach a grayscale level up to 80.

This method also performs well in the physiological range (35% to 50% hematocrit). The high luminous exposure range provides the best performance with an average R^2^ = 0.952. The average R^2^ values of the low and medium luminous exposure ranges are 0.848 and 0.875, respectively.

### 4.4. Determination of Hematocrit from Grayscale and Luminous Exposures

Matlab was used to develop a relationship between the grayscale value of the images captured during the blood flow experiments and the different luminous exposures and hematocrit values. Equation (2) describes the relationship between the average grayscale and hematocrit at different luminous exposure ranges.

(2) $$G=\frac{\alpha \cdot H_{v}+\beta }{\mathrm{Hct}+\gamma \cdot H_{v}+\delta }$$

Equation (3) is rearranged from Equation (2) to determine the hematocrit percentage value more directly.

(3) $$\mathrm{Hct}=\alpha \cdot \frac{H_{v}}{G}+\beta \cdot \frac{1}{G}-\gamma \cdot H_{v}-\delta$$

where G is the average grayscale, H_v_ is the luminous exposure in the unit of lux·µs, and Hct is the hematocrit in percentage. The α is the parameter of the ratio of luminous exposure to average grayscale in the unit of 1/lux·µs, β is the inverse affecting parameter, which is inversely proportional to the grayscale effect on the hematocrit percentage value, γ is the proportional affecting parameter of luminous exposure in the unit of 1/lux·µs and δ is the constant. The values of α, β, γ, and δ are listed in Table 1. It should be noted that the light source intensity and the exposure time of the camera could proportionally affect the H_v_ in the equation. The color temperature setting in the camera needs to be calibrated to the light source’s color temperature to ensure the accurate G value in the equation. 

Figure 6 and Figure 7 prove that the experimental data collected from the blood samples in this experiment fits well with the hematocrit–grayscale relationship proposed in Equation (3). These experimental data confirmed the reproducibility of the proposed optical hematocrit measurement method. All of the data points matched the equation within a reasonable error range (average R^2^ = 0.988), which confirmed the reliability of this method. In summary, these experimental results suggest that this optical quantification method is reproducible and reliable in a wide range of optical conditions. In this study, two donors’ blood containing anticoagulant K2-EDTA was used in experiments and achieved a good reproducibility. All the materials used in experiments are the most commonly used consumables: centrifuge tubes, microliter size pipette, tips, PDMS, glass slides, etc. The microscopy system used a halogen light, which is the most popular light source for the inverted microscope with transmitted light.

## 5. Conclusions

A novel optical hematocrit measurement method based on the microfluidic platform has been introduced in this study. It has been demonstrated that the luminous exposure at the camera sensor plays a critical role in this optical measurement method. To ensure measurement accuracy, some calibrations of the camera settings are required. Scientific methods were used to demonstrate the accuracy of this optical hematocrit measurement method. The data suggests the reliability and repeatability of this method. Since the inverted microscopy system and other materials used in this experiment are commonly equipped in most microfluidic laboratories, this proposed method can be applied to measure the hematocrit in various microfluidic platforms. Similarly, the application of this method is expected to be implemented to measure the concentration of specific components in other complex fluids. Even though this study used only blood samples from two donors and controlled the microchannel geometry, the principles of this method can be extended to develop measurement applications for a broader class of micro-scale complex fluids, especially the measurement of flow motion using a high-speed camera.

## Figures and Tables

**Table 1:** Table of Constants.

Hv (lux·µs)	α (1/lux·µs)	β	γ (1/lux·µs)	δ
Less than 6000	0.084	−83.1	0.0021	0.079
6000~15,000	0.10	−189	0.00070	5.7
Bigger than 15,000	0.15	−842	0.0014	−4.1

## Data Availability

Not applicable.
